# Clinical effects of combined anteversion and offset on postoperative dislocation in total hip arthroplasty

**DOI:** 10.1186/s42836-024-00245-3

**Published:** 2024-05-05

**Authors:** Ryo Hidaka, Kenta Matsuda, Shigeru Nakamura, Masaki Nakamura, Hirotaka Kawano

**Affiliations:** 1 Department of Orthopedic Surgery, Teikyo University School of Medicine, 2-11-1, Kaga, Itabashi-Ku, Tokyo, 173-8606 Japan; 2 Department of Orthopedic Surgery, Nishitokyo Chuo General Hospital, 2-4-19, Shibakubo-Cho, Nishitokyo, Tokyo, 188-0014 Japan; 3 Department of Orthopedic Surgery, Toranomon Hospital, 2-2-2, Toranomon, Minato-Ku, Tokyo, 105-8470 Japan

**Keywords:** Total hip arthroplasty, Dislocation, Combined anteversion, Offset

## Abstract

**Background:**

Implant impingement and soft tissue tension are factors involved in dislocation after total hip arthroplasty (THA). Combined anteversion (CA) has been used as an indicator for implant placement. However, optimal implant placement remains a challenge. Moreover, the effect of changes in offset on dislocation is still unclear. In this study, we aimed to clarify the effects of postoperative CA and pre- and postoperative changes in offset on dislocation.

**Methods:**

Included were patients who underwent primary cementless THA between 2013 and 2020. The mean values of CA and offset in the dislocation and non-dislocation groups were compared. The CA values within ± 10% of the recommended values were defined as good CA, and those outside the range were rated as poor CA. The dislocation rates were compared between the good and poor CA groups and between the groups with and without increased offset.

**Results:**

A total of 283 hips were included. The mean values of CA in the dislocation and non-dislocation groups were significantly different (*P* < 0.05). The dislocation rate was significantly lower in the good CA group (*P* < 0.05). The dislocation rates in the groups with and without increased total offset were 0.5% and 4.3%, respectively (*P* = 0.004). There were no dislocations in patients with good CA and increased offset.

**Conclusions:**

The dislocation rate was significantly lower when implants were placed within ± 10% of the recommended CA value. Our results suggest that dislocation can be avoided by placing the implant in the good CA range and considering the increase in total offset on the operative side.

## Background

Complications after total hip arthroplasty (THA) are known to include infection, loosening, fracture, and dislocation. Of these, dislocation represents one of the most serious complications that may require revision. Therefore, preventing dislocation is essential for THA [1]. Various factors are involved in the post-THA dislocation, including surgical, patient-, and implant-related factors [2, 3]. Patient factors include age, cerebral dysfunction, and range of motion [2, 4]; implant factors include head diameter and neck design [2, 4]; surgical factors include operative approach, implant and bony impingement, and soft tissue strain [5]. Several studies have demonstrated that implant impingement because of inadequate implant placement is the main cause of post-THA dislocation [6–8].

Combined anteversion (CA), the optimal combination of cup inclination, cup anteversion, and stem anteversion to maximize the range of motion and minimize the risk of cup-neck impingement to reduce the risk of dislocation, has been reported to be a useful index for implant positioning [9–11]. Moreover, the offset serves as an index for bony impingement and soft tissue tension [5, 12]. However, few reports have evaluated THA dislocations based on both CA and offset. Additionally, although differences in offset between the operative and contralateral sides have been reported, the pre- and postoperative offsets on the operative side have not been described. In this study, we aimed to determine if obtaining the desired CA and combined offset would lead to a reduction in hip instability in a consecutive series of hip arthroplasties.

## Methods

### Participants

Overall, this study included 427 hips in 379 consecutive patients who underwent primary cementless THA via the posterior approach from 2013 to 2020 at our institution. This retrospective case–control study was conducted in compliance with the Helsinki Declaration and was approved by the institutional ethics board (19–174). The patients provided written informed consent prior to participation. Patients who used non-flatliners (*n* = 65), those who could not undergo postoperative computed tomography (CT) for any reason (*n* = 37), and those who could not be followed for more than 1 year (*n* = 48) were excluded. All hips were followed up for at least 1 year, during which postoperative dislocations were recorded.

### Preoperative planning

Preoperative planning was performed using a three-dimensional (3D) template (ZedHip™ Lexi Co., Ltd., Tokyo, Japan) based on preoperative CT. For the pelvic coordinates, the functional pelvic plane was used as the reference plane passing through the bilateral anterior iliac spines and parallel to the CT table [13], and for the femoral coordinates, the retrocondylar plane was used as the reference plane passing through the posterior point of the greater trochanter and the posterior points of the medial and lateral femoral condyle [14]. The target inclination of the acetabular component (cup radiographic inclination) was set to 43° in all cases. Anteversion of the femoral component (stem anteversion) was predicted by placing a stem along the shape of the proximal femur using a preoperative 3D template. The target anteversion of the acetabular component (cup anatomical anteversion) was determined for each case using Yoshimine’s CA formula (cup anatomical anteversion + cup radiographic inclination + 0.80 × stem anteversion = 90.8°) [11].

### Surgical procedure and postoperative evaluation

All surgical procedures were performed in the lateral decubitus position, and a CT-based navigation system (Stryker CT-Hip System V1.1, Stryker-Leibinger GmbH & Co. KG, Freiberg, Germany) was used as the acetabular component. The posterior articular capsule and short external rotator muscles were repaired in all cases [15]. All surgeries were performed by either of the two senior authors.

**Fig. 1:**
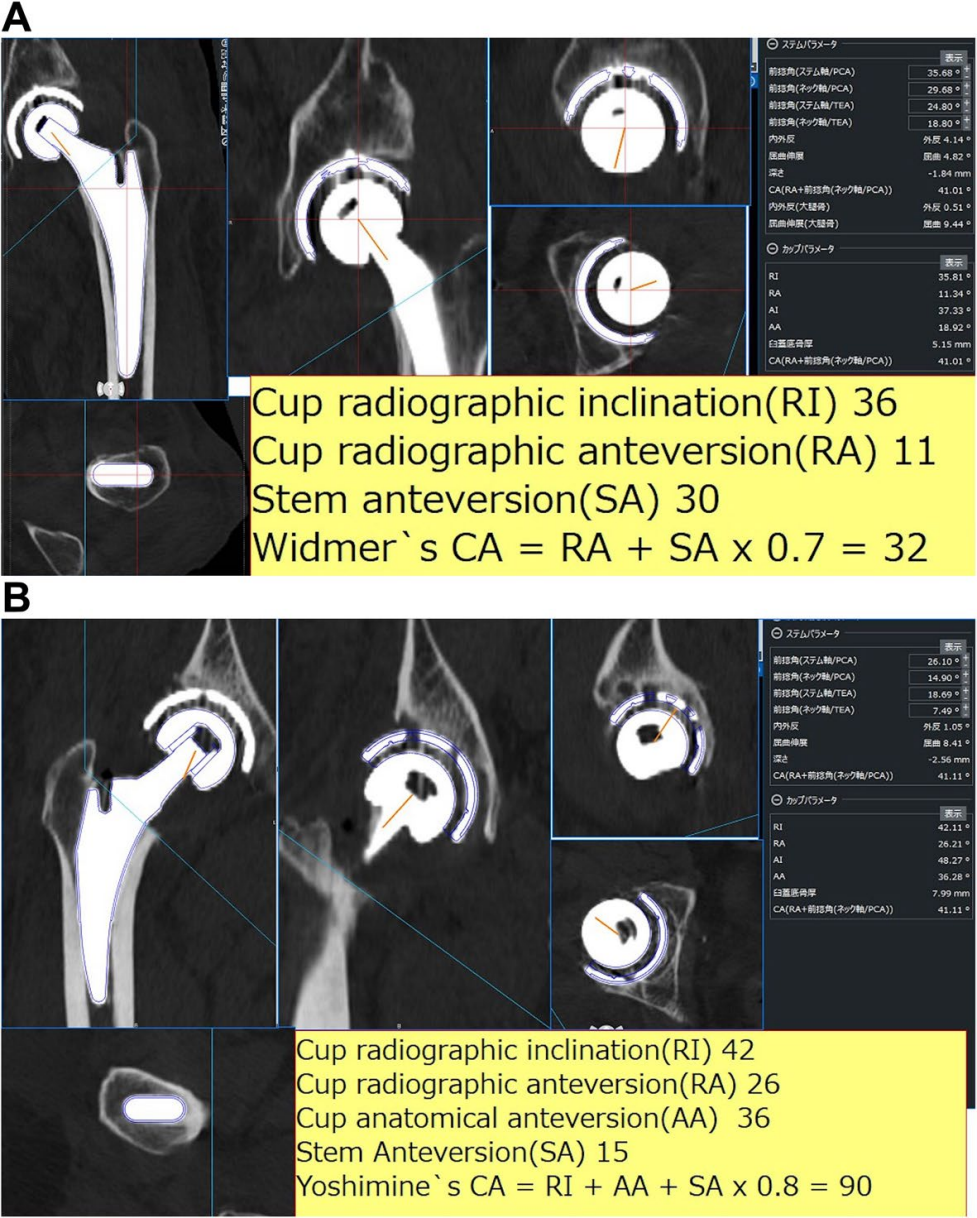
Measurement of the postoperative acetabular component and femoral component placement angles. **a** Widmer’s combined anteversion; **b** Yoshimine’s combined anteversion

A CT scan was performed at 1 week postoperatively as a routine protocol to evaluate implant placement and to confirm the presence of fractures. The postoperative CT data were imported into the template for 3D analysis. The postoperative placement angles of the acetabular and femoral components were measured using the template. Matching with the preoperative template data, a template of the same size as the actual implant was overlapped on the postoperative CT for measurement (Fig. 1a, b). Based on these placement angles, the postoperative CA values were calculated using the formulas of Widmer et al. (cup radiographic anteversion + 0.7 × stem anteversion = 37°) and Yoshimine et al. [10, 11]. The mean CA values of the dislocated and non-dislocated groups were calculated and compared. Cup radiographic inclination within 35°–55° and CA values within ± 10% of the recommended values (Widmer: 37° ± 4°, Yoshimine: 90.8° ± 9°) were defined as good CA, and those outside the range were defined as poor CA. The dislocation rates in the good and poor groups were compared. The absolute values of the difference between CA values calculated for each case and the recommended CA values using the formulas of Widmer et al. and Yoshimine et al. (37° and 90.8°, respectively) were calculated. Moreover, the cutoff values of CA for dislocation were examined. All offset measurements were made by projecting the distance on a horizontal plane using the template based on the preoperative and postoperative CT findings.

The horizontal distance from the pubic symphysis to the center of the femoral head was measured as the acetabular offset, and the horizontal distance from the center of the femoral head to a line passing through the center of the femoral shaft was measured as the femoral offset [16]. The sum of these offsets was defined as the total offset.

The mean values of the total, acetabular, and femoral offsets on the operated side were compared between the dislocated and non-dislocated groups. The difference between the preoperative and postoperative values of each of these three offsets was divided into the postoperative increase and non-increase groups, and their dislocation rates were compared. The number of dislocations in each combination of the total offset increased/non-increased group and the CA good/poor group was investigated. The dislocation rates were also compared in cases with inner heads < 32 mm and ≥ 32 mm.

### Statistical assessment

Demographic variables are described as medians and interquartile ranges for continuous data. All continuous variables were tested for normality using the Shapiro–Wilk test. We used the Mann–Whitney U-test to compare the means of non-normally distributed continuous variables and Fisher’s exact test to compare the proportions of categorical variables between the groups. Receiver operator coefficient (ROC) curves were created to calculate the cutoff value of CA for dislocation. The Youden method was used to calculate the optimal threshold scores to obtain the best balance between sensitivity and specificity [17]. All statistical analyses were conducted using JMP (version 15.1.0; SAS Institute Inc., Cary, NC, USA), with statistical significance defined as *P* < 0.05.

## Results

**Fig. 2:**
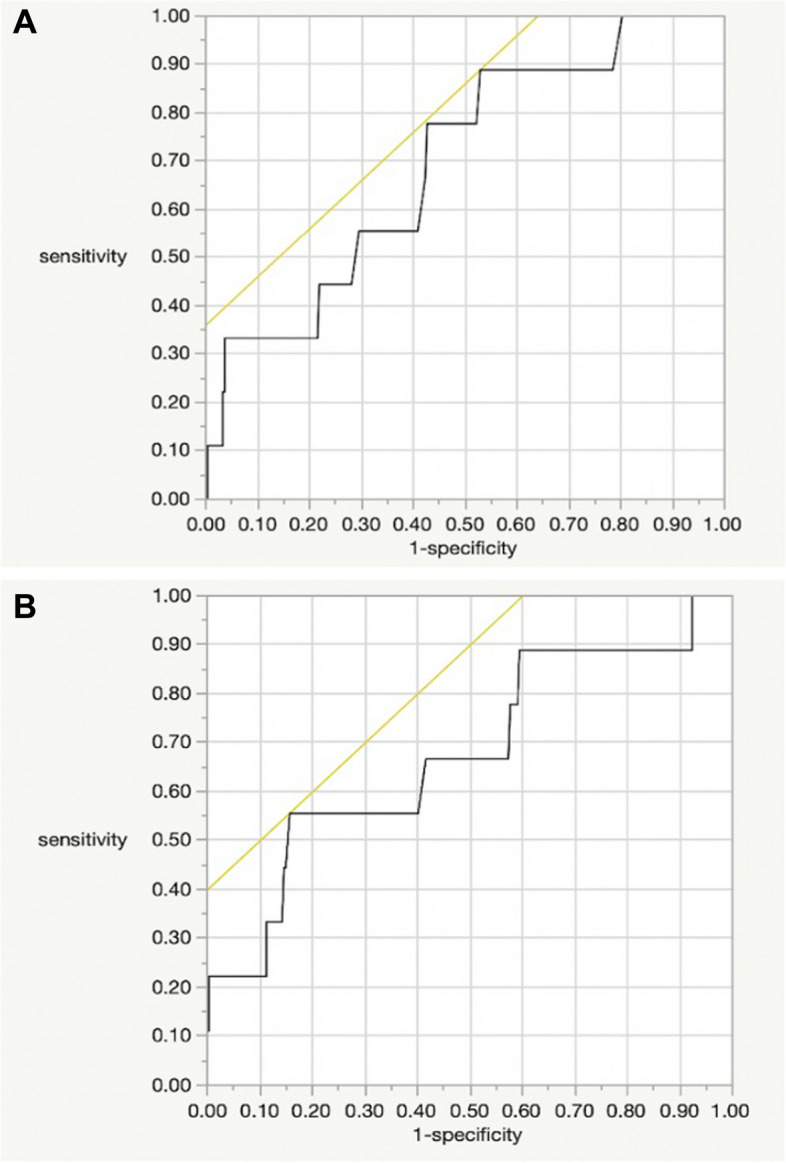
Receiver operating characteristic coefficient curve for dislocation. **a** Widmer (area under the curve: 0.696); **b** Yoshimine (area under the curve: 0.676)

All continuous variables were found to be non-normally distributed. The mean follow-up period lasted 5.3 ± 1.8 (2.5–9) years. Patient demographics are presented in Table 1. A total of 283 hips (256 patients) were included. There were nine dislocated hips (3.2%) and 274 non-dislocated hips. Dislocation was found in nine out of 238 cases treated by the S.N. and in zero out of 45 cases by K.M. There was no difference in the dislocation rates between the cases treated by the two surgeons (*P* = 0.363). The results of the univariate analysis comparing the dislocation and non-dislocation groups are presented in Table 2. A significant difference was observed between patients with a preoperative diagnosis of osteoarthritis and those without osteoarthritis. The mean CA values, as calculated by employing both Widmer et al.’s and Yoshimine et al.’s equations, were significantly different between the dislocation and non-dislocation groups. The good CA group included 158 hips, according to Widmer et al.’s study, and 233 hips, according to Yoshimine et al.’s study, with significantly lower dislocation rates in the good CA group. Based on the ROC curve analysis, the cutoff values for dislocation in Widmer’s CA and Yoshimine’s CA were 37° ± 3.3° and 90.8° ± 10.3°, respectively (Fig. 2a, b). The area under the ROC curve for Widmer’s CA was 0.696, the sensitivity was 0.889, the specificity was 0.471, and the Youden index was 0.36. For Yoshimine’s CA, the area under the ROC curve, sensitivity, specificity, and Youden index were 0.676, 0.556, 0.873, and 0.4, respectively. The mean total, acetabular, and femoral offsets on the operated side were not different between the dislocation and non-dislocation groups. The number of increased offsets was 169 (59.7%), 64 (22.6%), and 223 (78.8%) for total, acetabular, and femoral offsets on the operated side, respectively. Regarding the number of dislocations, the increase and non-increase groups were significantly different only in the total offset. The means of the preoperative and postoperative differences in offset were not significant between the dislocation and non-dislocation groups. When dislocation was evaluated using both total offset and CA and both equations, dislocations were observed in cases with poor CA or no increase in offset, but no dislocations were found in cases with good CA and increased offset (Table 3). The femoral heads used had sizes of 28, 32, and 36 mm. The femoral heads of 28 mm were used in 57 cases, of which three were dislocated, with no significant difference found in dislocation compared to cases of ≥ 32 mm.

**Table 1:** Patient demographics

Characteristic		Value
Number of patients (hips/cases)		283/256
Gender (hips/cases)	Male	68/58
	Female	215/198
Age at THA^a^ (years)		63 (55–72)
BMI (kg/m^2^)^a^		23.9 (21.3–26.9)
Diagnosis (hips/cases)	OA	216/196
	ONFH	51/44
	FNF	8/8
	RA	3/3
	RDC	5/5

*BMI* Body mass index, *OA* Osteoarthritis, *ONFH* Osteonecrosis of the femoral head, *FNF* Femoral neck fracture, *RA* Rheumatoid arthritis, *RDC* Rapidly destructive coxopathy, *THA* Total hip arthroplasty

^a^Values are presented as medians (interquartile ranges)

**Table 2:** Results of the univariate analysis of factors for dislocation after total hip arthroplasty

Variable		Dislocation ( +) *n* = 9	Dislocation (-) *n* = 274	*P-*value	*Chi-square* value
Age at THA* (years)		61.7 ± 14.2 (40–79)	63.4 ± 11 (33–85)	0.75	0.10
Sex (hips/cases)	Male	2/2	66/56	1.00	0.02
	Female	7/7	208/191		
BMI (kg/m^2^)*		25 ± 5.5 (17.2–36.6)	24.5 ± 4.4 (15.8–39.6)	0.76	0.10
Diagnosis (hips)	OA	4	212	0.037^a^	5.2
	Others	5	62		
	ONFH	2	49		
	FNF	1	7		
	RA	1	2		
	RDC	1	4		
Mean CA* (degree)	Widmer	30.4 ± 8.5 (15.1–41.2)	37.1 ± 5.9 (19.8–62.1)	0.017^a^	5.74
	Yoshimine	81.3 ± 12 (60–95)	90.6 ± 7.4 (66.7–118.7)	0.014^a^	5.98
Widmer CA (hips)	Good	2	156	0.047^a^	4.26
	Poor	7	118		
Yoshimine CA (hips)	Good	4	229	0.01^a^	9.17
	Poor	5	45		
Total offset (hips)	Increase ( +)	1	168	0.004^a^	9.13
	Decrease (-)	8	106		
Acetabular offset (hips)	Increase ( +)	2	62	1.00	0.001
	Decrease (-)	7	212		
Femoral offset (hips)	Increase ( +)	6	217	0.41	0.82
	Decrease (-)	3	57		
Total offset D* (mm)		-3.5 ± 8.0 (-15.9–12.7)	1.1 ± 8.9 (-31.7–43.4)	0.06	3.55
Acetabular offset D* (mm)		-6.1 ± 8.0 (-22.3–3.2)	-5.1 ± 6.6 (-30.8–26)	0.95	0.004
Femoral offset D* (mm)		1.6 ± 5.4 (-4.7–12.6)	5.3 ± 7.0 (-14.1–29.9)	0.08	3.04
Femoral head size	≥ 32 mm	6	220	0.39	1.01
	< 32 mm	3	54		

*BMI* Body mass index, *CA* Combined anteversion, *D* the value obtained by subtracting the preoperative value from the postoperative value for each offset, *THA* Total hip arthroplasty

^*^Values are presented as mean ± standard deviation (range)

^a^Statically significant with *P*-value < 0.05

**Table 3:** Dislocation rate of each group of combined anteversion and total offset (dislocated cases/total cases)

**Widmer**	**TO decrease**	**TO increase**
CA good	3.2% (2/62)	0% (0/96)
CA poor	11.5% (6/52)	1.4% (1/73)
**Yoshimine**	**TO decrease**	**TO increase**
CA good	4.8% (4/84)	0% (0/126)
CA poor	20% (4/20)	3.6% (1/28)

*CA* Combined anteversion, *TO* Total offset

## Discussion

The investigation was performed to determine the effect of CA and offset on dislocation after THA. Our findings revealed that the mean values of both Widmer’s and Yoshimine’s CA calculations significantly differed between the dislocation and non-dislocation groups, supporting the effectiveness of CA. While the optimal orientation of hip components in patients with primary osteoarthritis is suggested to be based on native hip orientation, only 63% of patients fell within the “safe” combined anteversion range of 20° to 40° in the anatomical reference frame [18]. Further complicating the relationship between THA component position and dislocation is the evidence that cup position within the Lewinnek safe zone does not protect against postoperative instability [19].

Numerous studies using navigation systems in THA have reported that discrepancies between preoperative target values and postoperative measured values of implant placement angle are inevitable [20–22]. According to these previous reports, it is difficult to place the cup precisely at the generally recommended combined anteversion angle. Moreover, Widmer et al. assume that the radiographic inclination of the cup should be in the range of 40°–45° for CA. However, it is also difficult to place the implant within this range. In this study, only 155 implants were placed within this range. Therefore, based on the aforementioned reports and considering possible CA errors in surgery, we defined good CA as a cup radiographic inclination within the range of 35°–55° and ± 10% of the recommended CA value. The results of the present study demonstrated that dislocation was significantly lower in the good CA group, and the cutoff values of CA for postoperative dislocation were 37° ± 3.3° and 90.8° ± 10.3° for Widmer and Yoshimine, respectively. This range is almost the same as our definition of good CA, and it covers the pre- and postoperative differences in implant placement angles in previous reports. Significantly fewer dislocations occurred when implants were placed within this range, which is useful as an indicator of dislocation prevention. However, as cases of dislocation occurred even when implants were placed within this range, it would be difficult to prevent dislocation after THA using CA alone as an indicator of implant placement.

CA has been reported to be effective only in the prevention of implant impingement but not in bony impingement [23]. Previous studies have shown the importance of not only implant impingement but also bony impingement and soft tissue tension in the prevention of dislocation after THA [5, 23, 24]. Studies on bony impingement have shown that increased acetabular offset provides an increased range of motion for flexion and internal rotation by decreasing the effect of impingement of the greater trochanter on the anterior acetabulum [25–27]. Increased femoral offset improves hip abductor strength by lengthening the functional lever arm, reducing impingement, and increasing postoperative joint stability [24, 28] and hip range of motion [29, 30]. Thus, post-THA dislocation is influenced not only by CA but also by acetabular offset and femoral offset, and all of these indicators should be considered during surgery. In this study, the results revealed no significant difference in the dislocation rate between the groups with and without increased acetabular and femoral offsets but a significant difference in the dislocation rate between the groups with and without increased total offset. In THA of patients with osteoarthritis, medialization by reaming is often necessary to place the acetabular component in an anatomically normal acetabulum [31]. The femoral offset should be increased to compensate for the decrease in acetabular offset due to the medialization. Thus, both offsets need to be evaluated [32], and avoiding a decrease in the total offset is important [33], which supports our results. In this study, the distance from the pubic symphysis to the femoral shaft was measured as the total offset. Therefore, both the femoral and acetabular offsets were used in the evaluation. In both Widmer’s and Yoshimine’s CA, there were dislocations under the condition of good CA alone but not in cases with good CA and increased total offset. This suggests that dislocation can be further avoided by considering not only CA to prevent implant impingement but also the increase in total offset on the operated side to avoid soft tissue hypotension and bony impingement.

Although a previous report showed no difference in the risk of dislocation between the increased and non-increased global offset groups [34], all previous reports on offset for dislocation after THA have compared the offset between the healthy and the operative sides. Krishnan et al. reported that the mean difference between the right and left sides of the normal hip offset was 2.54 ± 2.31 mm and that hip offsets were not always symmetrical, even in normal hips [35]. Additionally, determining the appropriate center of rotation of the hip joint in patients with bilateral morbidity or severe dysplasia is difficult [36]. In our study, we found a significant difference in the pre- and postoperative total offset changes on the operative side between the dislocation and non-dislocation groups. This method of evaluating offset has a great advantage in that it is not affected by the condition of the contralateral hip joint. Our findings also suggest that when postoperative offset on the operative side is reduced from the preoperative offset, soft tissue tension is reduced, resulting in bony impingement and increased susceptibility to dislocation. To our knowledge, no other study has compared the pre- and postoperative offsets on the affected side. In this study, with regard to the occurrence of pain on the lateral side of the hip, there was no significant difference between patients with postoperative femoral offset > 5 mm on the operated side compared with the healthy side and those with offset < 5 mm. Many studies have reported that pain does not increase with increasing offset, which supports our results [25, 37–40].

The limitations of this study should be noted. First, three types of femoral head sizes were used in this study. With respect to the effect of different head sizes on dislocation, many reports have shown that there was less dislocation in heads sized ≥ 32 mm compared to those sized 28 mm [41]. However, in this study, no difference in dislocation was observed after comparing head sizes of 28 and ≥ 32 mm. Similar results were obtained for CA and offset for dislocation only in cases with a head size of ≥ 32 mm. Second, we used a non-flat liner to prevent dislocation in patients with inadequate intraoperative stability. These cases were excluded from the study because postoperative CT could not accurately measure the angle of placement of the non-flat liner, which might overestimate the effect of CA on dislocation. Third, the retrospective nature of this study might introduce bias. Although a multivariate analysis is necessary to reduce bias, the small sample size of nine patients with dislocations in our study precluded such analysis. Future studies should aim to include larger sample sizes of dislocated cases and adopt a prospective design to enhance statistical validity. Fourth, while the ideal offset goal should be based on the unaffected contralateral side, this was not feasible in our study due to the high incidence of bilateral morbidities. Additionally, the long-term adverse effects in cases of excessively increased femoral offset were not evaluated. Polyethylene wear reportedly increases when femoral offset increases by more than 5 mm compared with the normal hip joint [42]. In this study, 22 cases had an increase in the femoral offset by ≥ 5 mm compared with the healthy side in 112 patients with unilateral disease. These patients need to be carefully monitored in the future. Fifth, although a review article indicated that several sagittal spinopelvic characteristics are related to THA dislocation [43], we were unable to assess spinopelvic parameters in our retrospective study due to the lack of available imaging data.

## Conclusions

The dislocation rate was significantly lower when implants were placed within ± 10% of the recommended CA value, suggesting that placement within this range can prevent postoperative dislocation. In addition, an increase in the total offset on the operative side may prevent more dislocations.

## Data Availability

The datasets used and/or analyzed during the current study are available from the corresponding author on reasonable request.

## Abbreviations

THA: Total hip arthroplasty
CA: Combined anteversion
ROC: Receiver operator coefficient
3D: Three-dimensional
